# Diagnostic Challenges in Autoimmune Pancreatitis: A Case of Progressive Painless Jaundice With an Uncertain Etiology

**DOI:** 10.7759/cureus.101544

**Published:** 2026-01-14

**Authors:** Idan Grossmann, Sidra Ahsan, Xiu Sun, Shuvendu Sen, Lee Peng

**Affiliations:** 1 Department of Internal Medicine, Hackensack Meridian Health Jersey Shore University Medical Center, Neptune City, USA; 2 Department of Gastroenterology and Hepatology, Hackensack Meridian Health Jersey Shore University Medical Center, Neptune City, USA; 3 Department of Pathology, Hackensack Meridian Health Jersey Shore University Medical Center, Neptune City, USA

**Keywords:** autoimmune pancreatitis (aip), autoimmune pancreatitis mimicker, diagnostic delay, igg4-related disease (igg4-rd), painless jaundice

## Abstract

Autoimmune pancreatitis (AIP) is a rare cause of pancreatitis compared with more common etiologies such as gallstone or alcohol-related pancreatitis. In this report, we present the case of a 61-year-old man with no significant past medical history who presented with progressive, painless jaundice. His non-specific clinical presentation was compatible with a broad differential diagnosis, initially raising concern for malignant biliary obstruction. Ultimately, endoscopic ultrasound with tissue biopsy was required to establish the diagnosis of AIP, highlighting the diagnostic challenges posed by this uncommon entity.

## Introduction

Autoimmune pancreatitis (AIP) is a distinct type of chronic pancreatitis, occurring in less than 1% of the general population, with a predilection for elderly males [1]. A nationwide study in Japan revealed a prevalence of 4.6-10.1 per 100,000 persons and an annual incidence of 1.4-3.1 per 100,000 persons, with a male-to-female ratio of approximately 3:1 and a mean age at diagnosis around 65-68 years [2].

Type 1 AIP is more common than type 2 AIP, which is less frequent, typically affects younger individuals, and is associated with inflammatory bowel disease [3]. The most common clinical presentation of AIP is painless obstructive jaundice; however, the presentation can be non-specific and diagnostically challenging [4].

We present a 61-year-old man with no prior medical history who developed gradually progressive jaundice. Initial history and diagnostic considerations favored malignancy, leading to significant diagnostic uncertainty. Due to the inconclusive presentation, confirmation of the diagnosis required a biopsy. This case highlights the importance of maintaining a high index of suspicion for AIP, as it remains a rare but critical diagnosis to consider.

## Case presentation

A 61-year-old male with no known past medical history presented with progressive painless jaundice for approximately six weeks, associated with dark urine, pale stools, and intermittent nausea, without identifiable aggravating or alleviating factors. He denied fever, chills, chest pain, shortness of breath, abdominal pain, unintentional weight loss, pruritus, or changes in bowel habits. He also denied alcohol or tobacco use and had no personal or family history of liver disease or malignancy.

**Figure 1:**
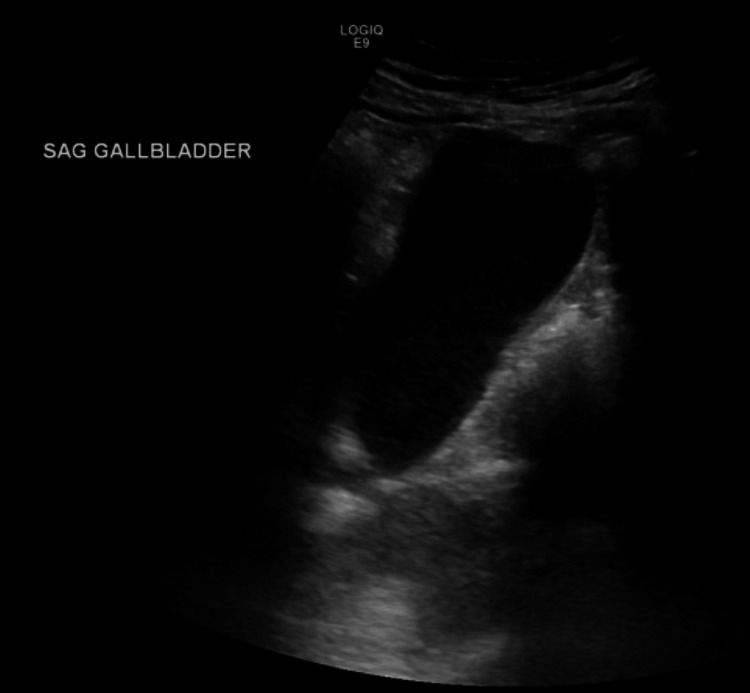
Right upper quadrant ultrasound demonstrating a distended gallbladder without gallstones or inflammatory changes.

**Figure 2:**
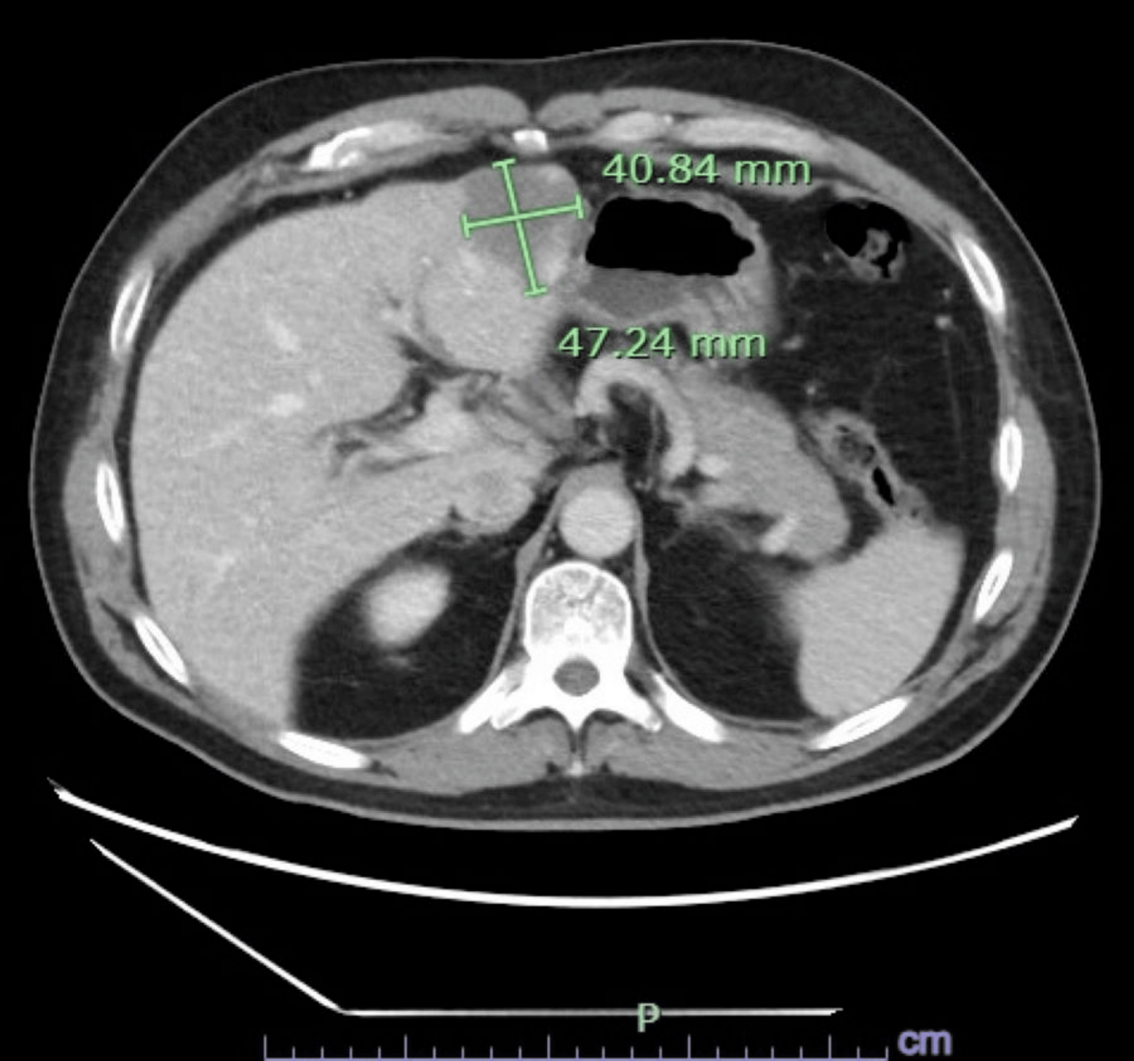
Contrast-enhanced CT of the abdomen demonstrating a hepatic lesion concerning for malignancy and a subtle nodular liver contour suggestive of early cirrhosis.

**Figure 3:**
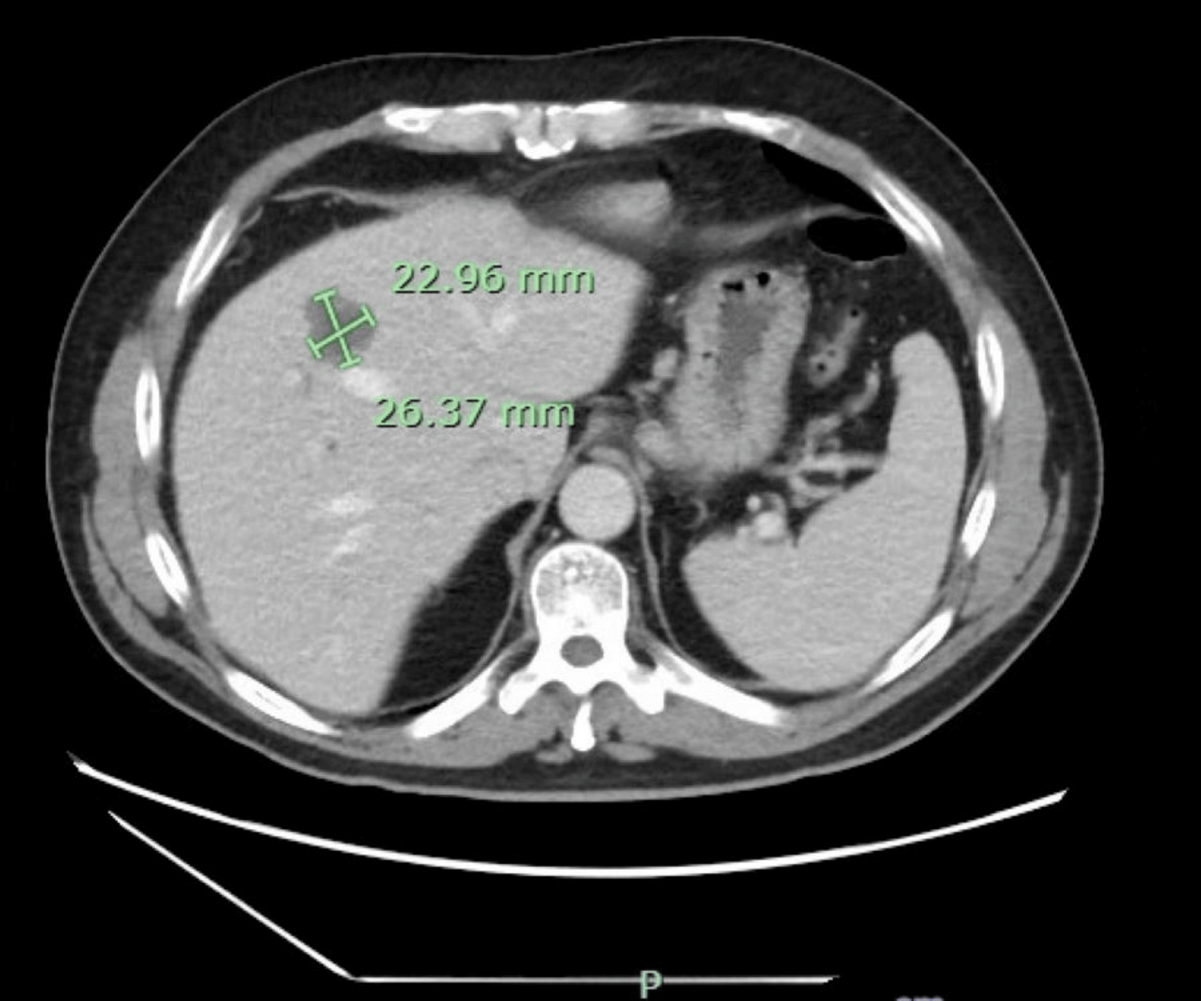
Contrast-enhanced CT of the abdomen at a different slice demonstrating a second hepatic lesion concerning for possible malignancy, with a subtle nodular liver contour suggestive of early cirrhosis.

**Figure 4:**
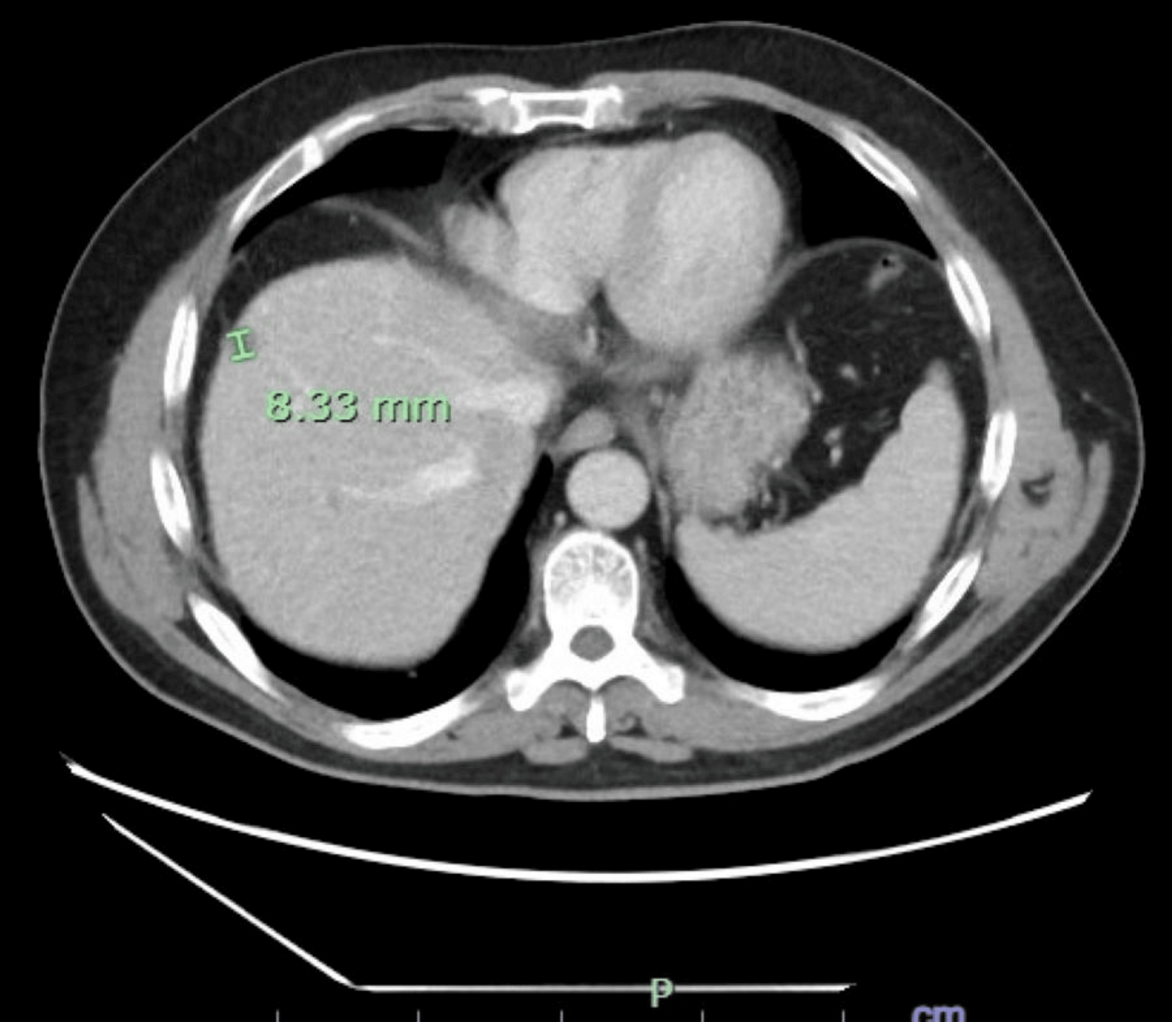
Additional abdominal CT slice demonstrating a third hepatic lesion and nodular liver contour.

Two weeks prior to the current presentation, the patient had been evaluated for similar symptoms. At that time, laboratory testing revealed a total bilirubin of 6.2 mg/dL. Right upper quadrant ultrasound demonstrated a distended gallbladder without gallstones and heterogeneous hepatic echogenicity (Figure 1). Contrast-enhanced computed tomography (CT) of the abdomen was obtained to evaluate painless jaundice with concern for a pancreatic mass; it was not performed using a dedicated triphasic liver protocol and showed three indeterminate hepatic lesions concerning for hemangiomas versus malignancy, mild gallbladder distention without biliary dilation, and contour changes possibly suggestive of early cirrhosis (Figures 2-4). The patient elected discharge after shared decision-making with plans for close follow-up and strict return precautions. However, due to a rapid rise in bilirubin on outpatient follow-up, the patient was referred back to the emergency department for further evaluation.

On presentation, he was afebrile and hemodynamically stable. Physical examination was notable only for scleral icterus and diffuse jaundice, without abdominal tenderness, hepatosplenomegaly, or stigmata of chronic liver disease.

Laboratory studies demonstrated a cholestatic pattern of liver injury, with a total bilirubin of 17.5 mg/dL, alkaline phosphatase 488 U/L, aspartate aminotransferase (AST) 204 U/L, alanine aminotransferase (ALT) 446 U/L, and a markedly elevated gamma-glutamyl transferase (GGT) of 1,378 U/L. Ferritin was elevated at 1,257 ng/mL with low serum iron levels of 32 µg/dL. Cancer antigen 19-9 (CA 19-9) and alpha-fetoprotein (AFP) were within normal limits, 18 U/mL and 2.7 ng/mL, respectively (Table 1).

**Table 1:** Initial laboratory results on presentation.

Laboratory Test	Result	Reference Range
White Blood Cell (WBC)	6.1 ×10^3^/µL	4.0-10.0 ×10^3^/µL
Hemoglobin (Hgb)	13.2 g/dL	13.0-17.0 g/dL
Platelets (Plt)	229 ×10^3^/µL	150-400 ×10^3^/µL
Sodium (Na)	137 mmol/L	135-145 mmol/L
Potassium (K)	4.2 mmol/L	3.5-5.1 mmol/L
Total Bilirubin	17.5 mg/dL	0.2-1.2 mg/dL
Alkaline Phosphatase (ALP)	488 U/L	40-129 U/L
Aspartate Aminotransferase (AST)	204 U/L	10-40 U/L
Alanine Aminotransferase (ALT)	446 U/L	7-56 U/L
Gamma-Glutamyl Transferase (GGT)	1,378 U/L	9-48 U/L
Ferritin	1,257 ng/mL	30-400 ng/mL
Serum Iron	32 µg/dL	60-170 µg/dL
Creatinine	0.9 mg/dL	0.7-1.3 mg/dL
Alpha-Fetoprotein (AFP)	2.7 ng/mL	<10 ng/mL
Cancer antigen 19-9 (CA 19-9)	18 U/mL	<37 U/mL

Serum IgG4 was markedly elevated at 757 mg/dL. Viral hepatitis serologies and Epstein-Barr virus testing were negative. Autoimmune serologies, including antinuclear antibody (ANA), anti-smooth muscle antibody (ASMA), antimitochondrial antibody (AMA), and perinuclear anti-neutrophil cytoplasmic antibodies (p-ANCA), were also negative (Table 2).

**Table 2:** Additional serologic and immunologic results on presentation

Test	Result
Hepatitis A IgM	Negative
Hepatitis B surface antigen (HBsAg)	Negative
Hepatitis B core antibody (anti-HBc IgM)	Negative
Hepatitis C antibody	Negative
Epstein-Barr virus (EBV) IgM	Negative
Antinuclear antibody (ANA)	Negative
Anti-smooth muscle antibody (ASMA)	Negative
Antimitochondrial antibody (AMA)	Negative
Perinuclear anti-neutrophil cytoplasmic antibodies (p-ANCA)	Negative
IgG4	757 mg/dL

**Figure 5:**
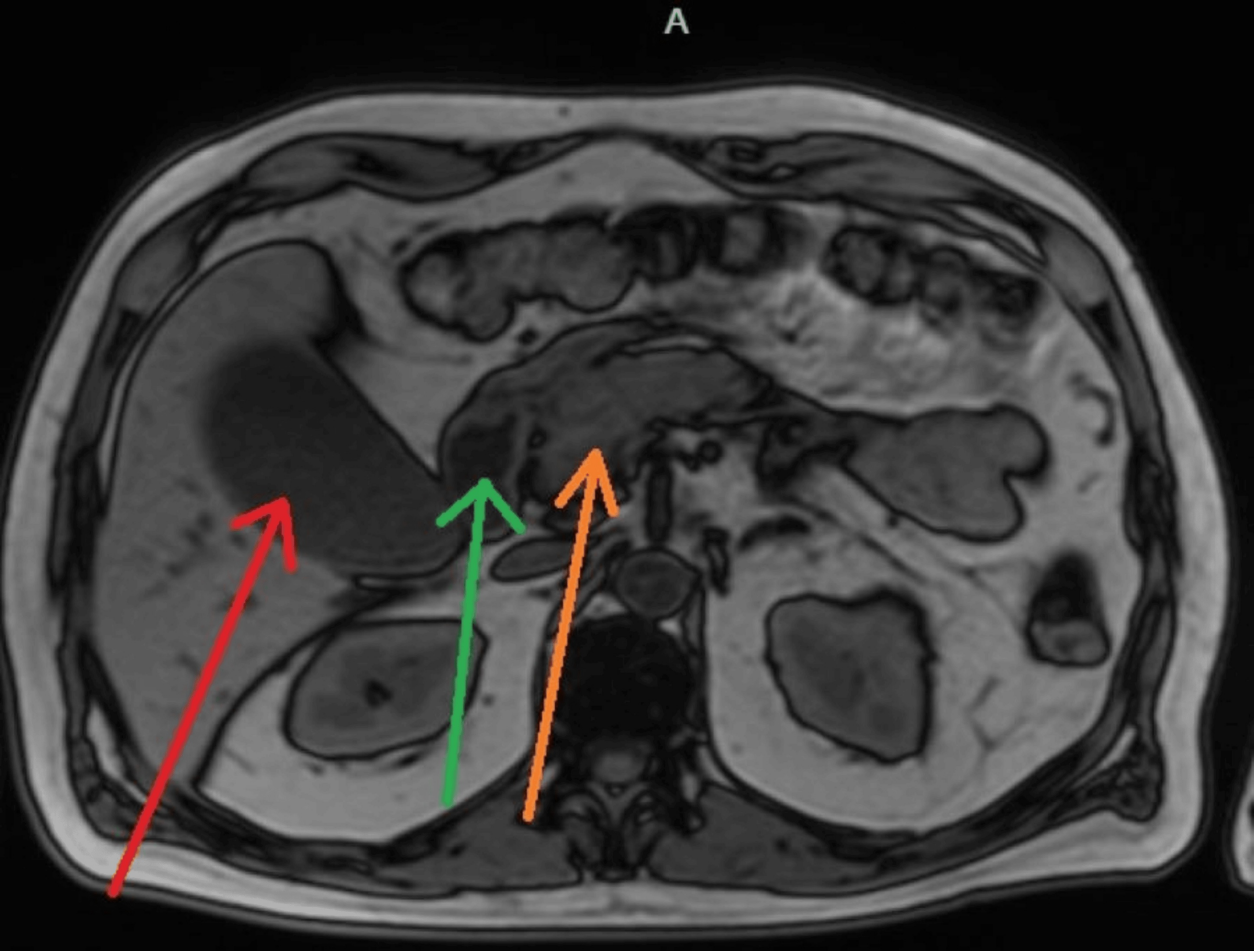
MRI of the abdomen with MRCP demonstrating a distended gallbladder (red arrow), moderately dilated proximal common bile duct (green arrow), and diffuse pancreatic enlargement with mild edema (orange arrow), without choledocholithiasis, discrete pancreatic mass, or pancreatic ductal dilation, findings suggestive of autoimmune pancreatitis.

**Figure 6:**
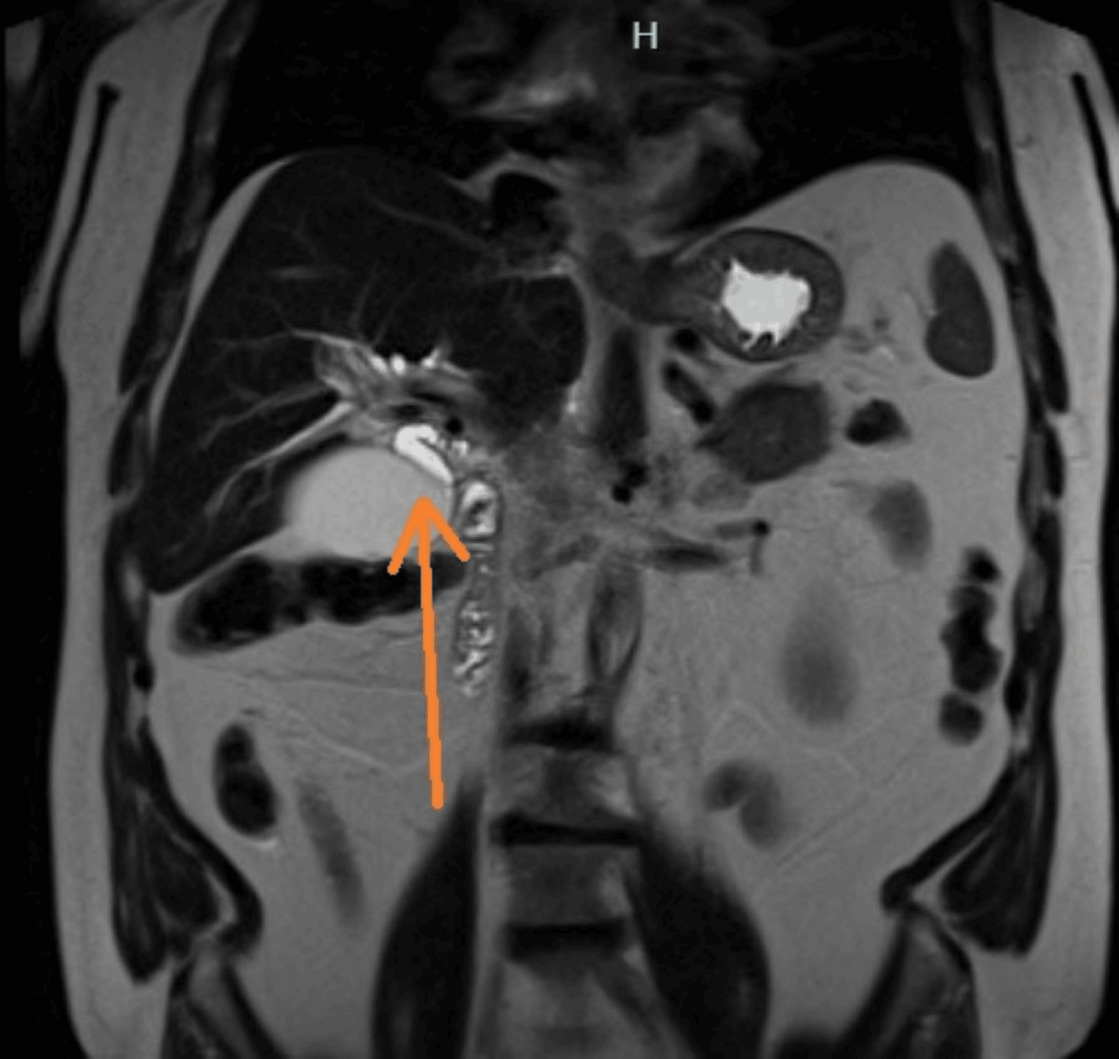
Coronal MRI of the abdomen with MRCP demonstrating a distended gallbladder and a moderately dilated proximal common bile duct (orange arrow).

Magnetic resonance imaging (MRI) of the abdomen with magnetic resonance cholangiopancreatography (MRCP) demonstrated a distended gallbladder with sludge but no cholelithiasis, mild-to-moderate intrahepatic biliary ductal dilation, and dilation of the proximal common bile duct to 8 mm with abrupt tapering at the level of the pancreatic head (Figures 5-6). The pancreas appeared diffusely enlarged with mild surrounding edema, without a discrete mass or pancreatic duct dilation. The previously identified hepatic lesions were consistent with hemangiomas. Given the findings of obstructive jaundice, biliary ductal dilation, diffuse pancreatic enlargement, and elevated IgG4 levels, AIP was strongly suspected.

The patient subsequently underwent endoscopic ultrasound (EUS), which demonstrated diffuse pancreatic enlargement without a focal mass, and a fine-needle biopsy of the pancreas was performed. Endoscopic retrograde cholangiopancreatography (ERCP) revealed a distal biliary stricture at the level of the pancreatic head, and a common bile duct stent was placed to relieve biliary obstruction.

**Figure 7:**
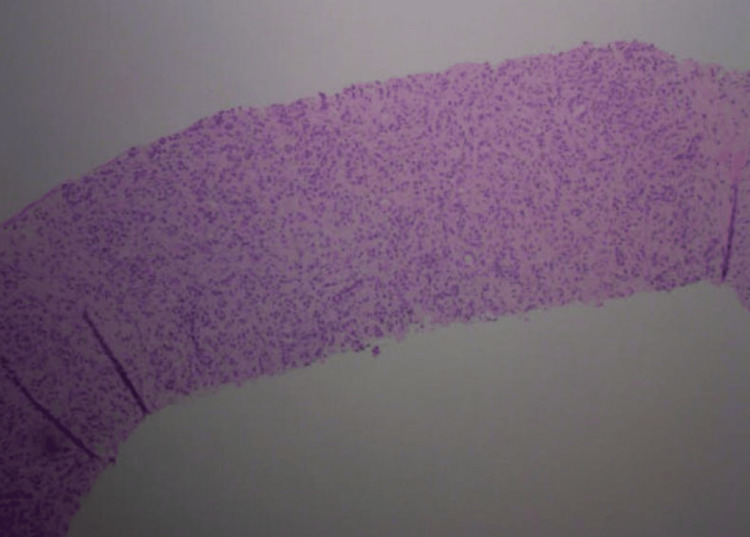
Low-power view of the pancreatic head biopsy showing pancreatic parenchyma with marked acinar atrophy, dense lymphoplasmacytic infiltrates, and stromal fibrosis.

**Figure 8:**
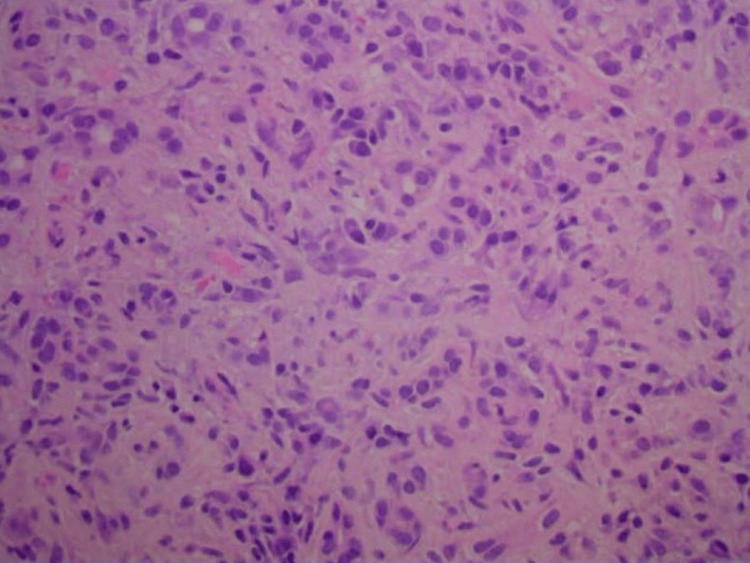
High-power view of the pancreatic head biopsy showing scattered ducts and acinar atrophy in a background of lymphocytes, plasma cells, and stromal fibrosis.

**Figure 9:**
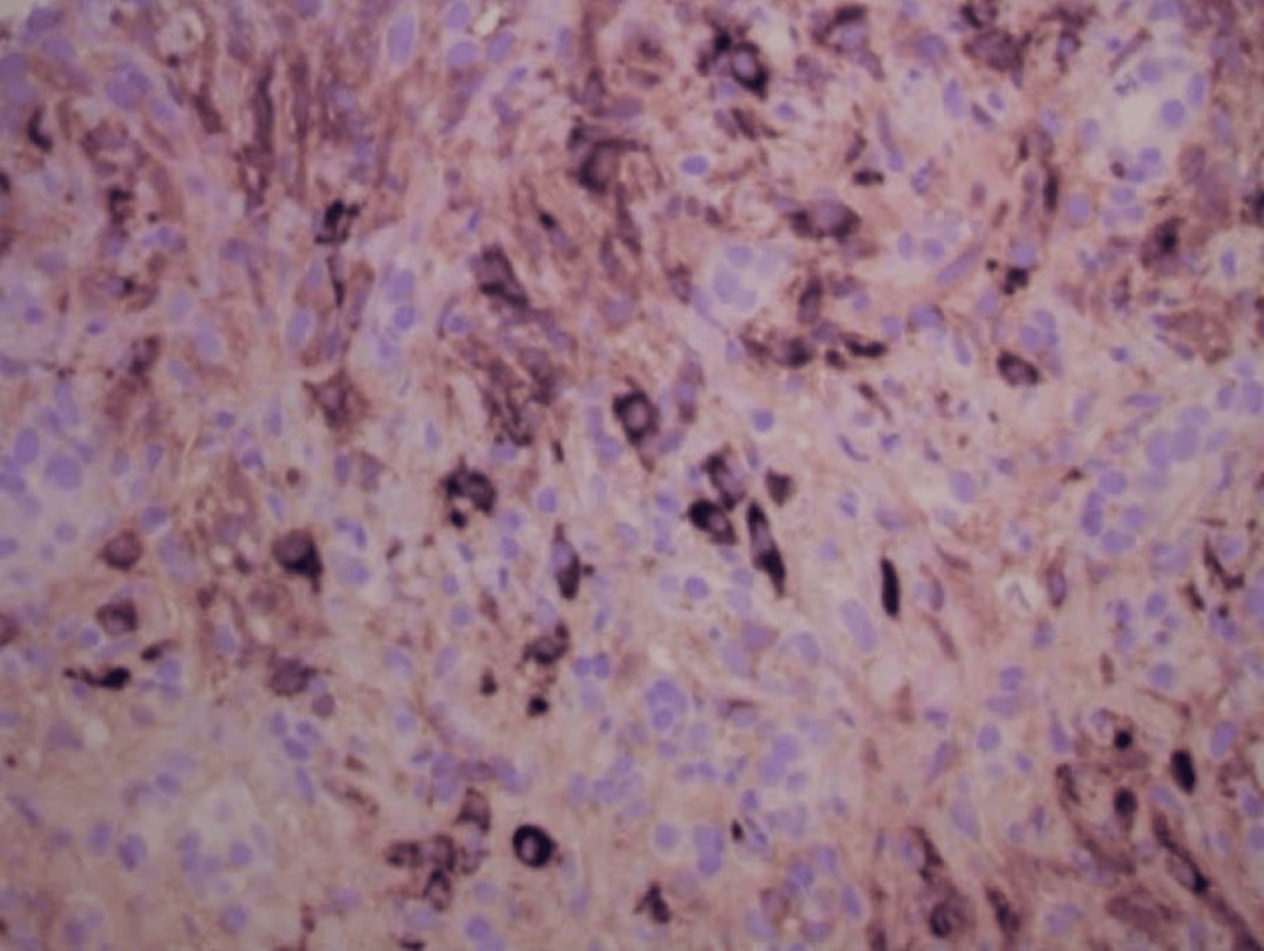
IgG4 immunostain demonstrating IgG4-positive plasma cells.

**Figure 10:**
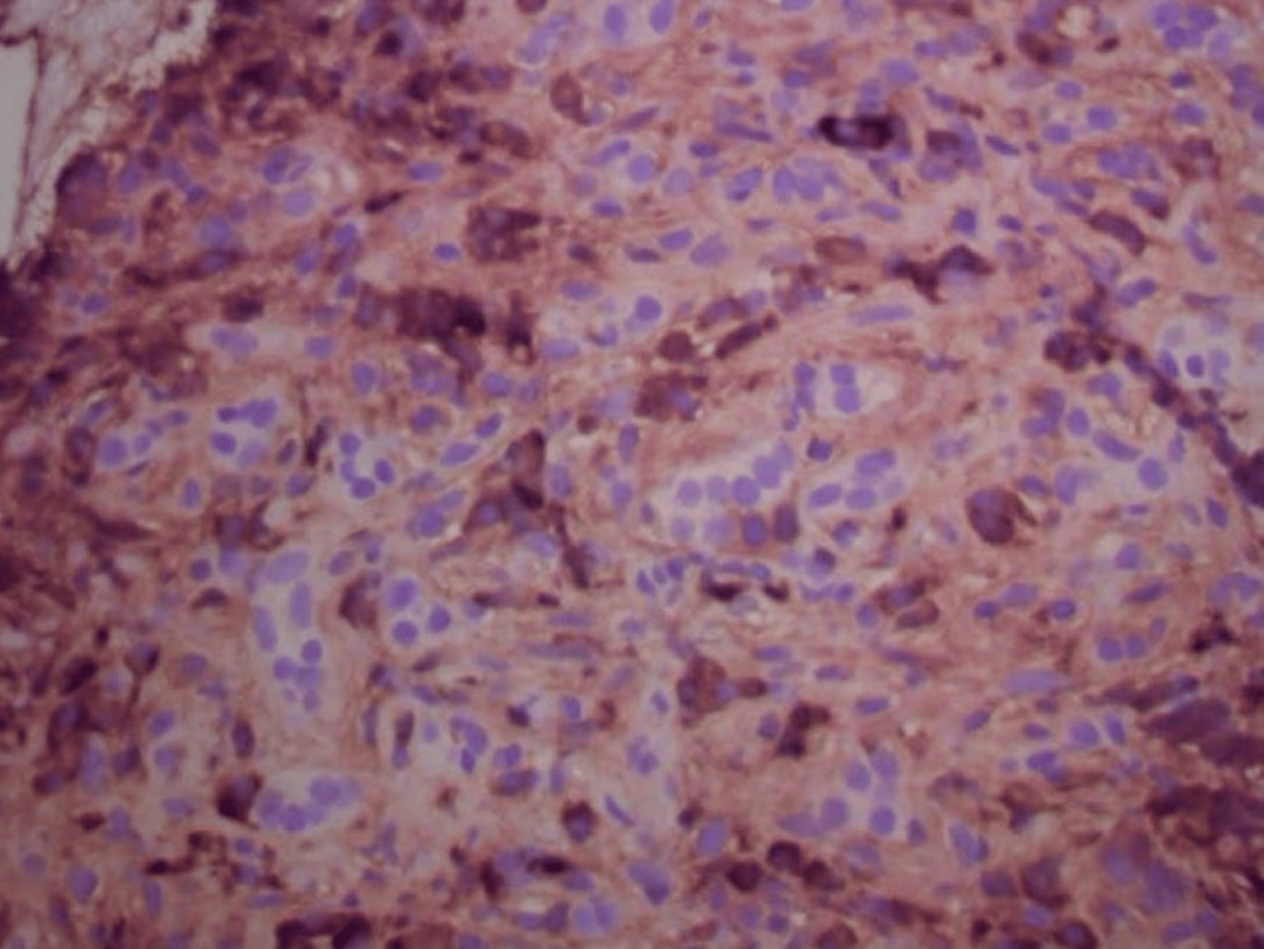
IgG immunostain demonstrating numerous scattered IgG-positive plasma cells.

Histopathologic examination of the pancreatic biopsy revealed lymphoplasmacytic infiltrates, scattered eosinophils, acinar atrophy, and fibrosis. Immunohistochemical staining was negative for carcinoma. Notably, immunostaining showed abundant IgG4-positive plasma cells, with more than 50 IgG4-positive cells per high-power field, consistent with IgG4-related AIP (Figures 7-10).

The patient’s hospital course was uncomplicated, with clinical improvement and downtrending liver enzymes following biliary decompression. He was discharged in stable condition and initiated on oral corticosteroid therapy. Outpatient follow-up was arranged, including repeat ERCP in three months to reassess the biliary stricture and manage the biliary stent.

## Discussion

AIP is an uncommon inflammatory condition with a highly variable clinical presentation. Manifestations range from subclinical findings to non-specific symptoms such as abdominal pain, reported in approximately 35% of cases, changes in bowel habits, and weight loss, to the classical presentation of painless jaundice, which occurs in up to 63% of patients [4]. Pancreatic exocrine and endocrine insufficiency can occasionally be observed in AIP [5]. In our case, the higher pretest probability of malignancy, particularly in the setting of inconclusive imaging, may have contributed to delayed diagnosis and treatment. This underscores the importance of considering AIP in diagnostically uncertain presentations.

The diagnosis of AIP can be challenging due to the relative rarity, the variable presentation, and the non-specific radiologic and serologic modalities that can overlap with different types of cancer and forms of pancreatitis. AIP typically presents with painless jaundice and weight loss, a presentation that overlaps with pancreatic adenocarcinoma. In addition, there may be overlap in imaging findings between AIP and pancreatic cancer. AIP typically demonstrates irregular narrowing of the main pancreatic duct, diffuse pancreatic enlargement, a capsule-like low-attenuation rim, and delayed enhancement on imaging [6,7]. Our case demonstrated diffuse pancreatic enlargement, distal common bile duct stricture with proximal biliary dilation, and no discrete pancreatic mass, consistent with AIP but also potentially mimicking malignancy. Moreover, fine-needle aspiration biopsy might yield false positives for malignancy, although this is rare. Deshpande et al. reported three false-positive cytologic diagnoses among 16 patients with AIP, including adenocarcinoma, solid-pseudopapillary tumor, and mucinous neoplasm [8]. Serum IgG4 is a supportive marker for type 1 AIP; however, this marker is positive in only 37% of cases and is normal in nearly all cases of type 2 AIP. Therefore, diagnosis of AIP requires a collection of clinical features, imaging, serology, histology, and response to steroids [9]. Due to the uncertainty involved in those cases, as in our case, the diagnosis and the initiation of treatment were delayed.

Biopsy is used as a diagnostic tool in approximately 30% of AIP cases and is typically reserved for patients in whom noninvasive diagnostic modalities are inconclusive [10]. The first-line treatment is steroids, which induce remission in the vast majority of cases. The necessity of biliary stenting in AIP remains debated, as many patients demonstrate rapid resolution of biliary obstruction with steroids alone. Recent evidence by Zhang et al. suggests that adjunctive endoscopic biliary drainage may not provide additional benefit beyond glucocorticoid therapy in IgG4-related pancreatobiliary disease [11]. In our patient, a stent was placed due to significant obstruction and symptomatic jaundice, but subsequent improvement with steroids highlights that corticosteroids remain the mainstay of therapy, and stenting is reserved for selected cases with persistent or severe obstruction. Relapse occurs more frequently in type 1 (approximately 30-60%) than in type 2, in which relapse is uncommon [12].

This case illustrates the diagnostic complexity of AIP and highlights the importance of utilizing multimodal diagnostic tools to achieve early detection, as timely diagnosis and treatment are associated with improved prognosis and prevention of complications.

## Conclusions

AIP is a rare and diagnostically challenging condition that can closely mimic pancreatic malignancy, particularly with painless obstructive jaundice and inconclusive imaging. A multimodal diagnostic approach, including imaging, serology, endoscopy, and histopathology, is often required. Early recognition and timely corticosteroid therapy, along with management of biliary obstruction, prevent complications and improve outcomes. It is important to educate the patient about the disease and treatment, including the components of therapy, potential side effects, and to engage in shared decision-making. The main limitation of this case is the diagnostic challenge in differentiating AIP from pancreatic cancer. Although AIP cannot currently be prevented, early consideration of IgG4-related disease facilitates prompt diagnosis and treatment.
